# Chloride Ion-Induced Modification of Passive Film on the Surface of 18%Ni High-Strength Steel

**DOI:** 10.3390/ma19020444

**Published:** 2026-01-22

**Authors:** Shule Yu, Boheng Yan, Botao Jiang, Hao Guo, Eshov Bakhtiyor, Liang Wang

**Affiliations:** 1 School of Materials Science and Engineering, Harbin University of Technology, Harbin 150001, China; 24s109290@stu.hit.edu.cn (S.Y.); 25b309029@stu.hit.edu.cn (B.Y.); wliang1227@hit.edu.cn (L.W.); 2 Zhengzhou Research Institute, Harbin Institute of Technology, Zhengzhou 450000, China; 3 Center for Research of Innovative Technologies, National Academy of Science of Tajikistan, Dushanbe 734025, Tajikistan; ishov1967@mail.ru

**Keywords:** martensitic steel, corrosion behavior, impedance spectroscopy, Tafel extrapolation, chloride ion concentrations

## Abstract

This work investigates the corrosion behavior of 18%Ni high-strength steel (00Ni18Co-8Mo5TiAl, solution-treated at 820 °C for 3 h and aged at 480 °C for 3 h) in NaCl solutions with 1%, 3.5%, and 6% chloride ions, as well as chloride ions’ effect on passive film properties. The corrosion process was systematically studied via chemical immersion tests (GB/T 17897-1999, 144 h, solution-to-sample contact area ratio 20:1) and electrochemical methods, including EIS (frequency range: 100 kHz–0.01 Hz) and Tafel polarization curves (scan rate: 10 mV/min). Passive film evolution was analyzed via Mott–Schottky curves (fixed frequency: 1000 Hz, scanning potential: −1 V to 1 V vs. SCE). Microstructural observations show the steel exhibits pitting corrosion in chloride environments, with corrosion products transforming from loose outer α-FeOOH/γ-FeOOH to dense inner Fe_3_O_4_/β-FeOOH. These dense products inhibit anodic reactions. Electrochemical results reveal polarization resistance decreases and corrosion current density rises with increasing chloride concentration. Mott–Schottky curves indicate that flat band potential increases from −0.2177 V to −0.1258 V with rising chloride concentration, increasing point defects in the passive film and weakening its self-healing ability.

## 1. Introduction

Martensitic high-strength steels have been widely employed in manufacturing engine blades as force-bearing members due to their good comprehensive ability [1,2]. Among them, 18%Ni series maraging steel is one of their representative steels. The high strength and toughness of steel can be obtained after simple heat treatment, but it is often affected by corrosion when stored in a chloride ion environment [3,4]. Investigations of corrosion behavior in different chloride ion concentration environments are of great significance for their practical application [5,6,7,8,9]. Studies have shown that the corrosion type of 18%Ni high-strength steels is mainly pitting corrosion, and the corrosion rate will accelerate in a chloride ion environment [10,11]. Li HY et al. [12] found that the chloride ion concentration has a certain effect on the corrosion of steel, and Sunaba T et al. [13] found that this effect is particularly significant in martensitic steel. Zhang Z et al. [14] noted that the local corrosion current density of steel will gradually increase with the increase of chloride ion concentration. Li D.G et al. [15] indicated that with the increase of chloride ion concentration, the anti-corrosion effect of the passive film on the substrate decreases. RF Tobias et al. [16] found that the passivation behavior of 18% nickel grade 250 martensitic aging steel is greatly influenced by the concentration of chloride ions. The Mott–Schottky curve is the main method used in the analysis of metal passive films, which is used to study the semiconductor properties of passive films, defect structures, and their relationship with corrosion behavior [17,18,19]. Previous reports have indicated that the corrosion product in the former stage is the mixture α-FeOOH and γ-FeOOH, which is orange, and then the corrosion product transforms the black ones, which are composed of Fe_3_O_4_ [20,21]. Simultaneously, some papers have found that the corrosion product will contain β-FeOOH when steel is in a chloride ion environment [22]. The corrosive effect of the medium in which chloride ions are located on materials has drawn increasing attention. Many studies have shown that the corrosion behavior of materials varies in different chlorine-containing media [23,24,25]. But, there are few reports on the effect of different chloride ion concentrations on the corrosion behavior and passive film and the changes of corrosion in different chloride media of 18%Ni high-strength steel.

Consequently, this paper takes 00Ni18Co8Mo5TiAl steel as the research object, makes use of the NaCl solution as the corrosion medium, and utilizes chemical immersion tests to study the corrosion behavior of 18%Ni high-strength steel in a chloride ion environment, combined with electrochemical testing methods to analyze and research chlorine-induced modification of passive film. The effect of different chloride ion concentrations on the corrosion behavior of 18%Ni high-strength steel provides a theoretical reference for corrosion research on 18%Ni high-strength steel in a marine environment.

## 2. Materials and Methods

### 2.1. Materials

The experimental material is forged 00Ni18Co8Mo5TiAl high-strength steel (equivalent to 18Ni (300), martensitic maraging steel). Its main chemical composition (mass fraction, %) is listed as follows: C 0.03, Ni 18.2, Co 8.3, Mo 5.1, Ti 0.4, Al 0.02, and Fe (balance). The steel was subjected to solution treatment at 820 °C for 3 h [26], followed by aging at 480 °C for 3 h [27], which is a typical heat treatment process for martensitic maraging steel to achieve high strength and toughness.

Specimens for chemical immersion tests had dimensions of 30 mm × 30 mm × 2 mm and were ground with silicon carbide sandpaper up to 1500 grit. Meanwhile, samples for electrochemical measurements were fabricated with dimensions of 10 mm × 10 mm × 2 mm.

For electrochemical samples, an exposed surface area of 1 cm^2^ was reserved as the working surface. An insulated copper wire was welded to the back of each specimen, and all surfaces except for the working surface were encapsulated with epoxy resin. Electrical resistance measurements between the working surface and the copper wire were performed to ensure overall conductivity, avoiding test failure caused by poor conductivity of the electrochemical samples.

For the preparation of NaCl solutions used in the tests, 10.1 g, 36.3 g, and 63.8 g of analytical-grade NaCl (purity ≥ 99.5%) were accurately weighed using a calibrated electronic balance (accuracy: 0.1 mg) for the 1%, 3.5%, and 6% (mass fraction) concentrations, respectively. Each weighed NaCl sample was dissolved in an appropriate volume of deionized water under magnetic stirring and then diluted to a final volume of 1 L in volumetric flasks to ensure concentration accuracy.

### 2.2. Chemical Immersion Test

The solutions used in the chemical immersion test were NaCl solutions with varying chloride ion concentrations (1%, 3.5%, 6% by mass fraction) and a 6% FeCl_3_ solution, which was prepared by dissolving 100 g of FeCl_3_·6H_2_O in 900 mL of 0.05 mol/L HCl solution to simulate an aggressive pitting corrosion environment.

The test setup is illustrated in Figure S1a. In accordance with the accelerated corrosion test standard [28], the samples were fixed horizontally on a custom polyvinyl chloride (PVC) support. A number of small holes were drilled on the side of the support to facilitate solution convection. The ratio of the solution volume to the sample surface area was strictly maintained at 20:1 to ensure uniform solution renewal around the sample. The immersion duration was set to 144 h. During the experiment, special attention was paid to the morphology of corrosion products formed on side A and side B of the samples, where side A refers to the side facing the container opening and side B refers to the side facing the container bottom.

### 2.3. Electrochemical Measurements

Electrochemical measurements were performed using a CHI660E potentiostat/galvanostat (CH Instruments, Shanghai, China) integrated with a frequency response analyzer, following a standard three-electrode configuration. All tests were conducted at 25 ± 2 °C in a thermostatic water bath (model: HH-601, Jintan Medical Instrument Factory, Jintan, Jiangsu, China) to maintain consistent temperature with the chemical immersion test. The electrochemical test apparatus is shown in Figure S1b. In the three-electrode cell, a saturated calomel electrode (SCE) served as the reference electrode (RE), a platinum plate as the counter electrode (CE), and the 18%Ni steel specimen as the working electrode (WE). The working surface of the WE was fully submerged ~10 mm below the solution surface and oriented toward the platinum CE. Prior to formal measurements, the specimens were immersed in the test solution for 60 min to stabilize the open-circuit potential (OCP).

Potentiodynamic polarization curves were scanned from −0.6 V to +1.0 V (vs. OCP) at a rate of 10 mV/min, in compliance with ASTM G5. Corrosion parameters (corrosion potential $E_{\mathrm{corr}}$ and corrosion current density $J_{\mathrm{corr}}$) were calculated via Tafel extrapolation using ZView3.1 software.

EIS measurements were performed after OCP stabilization, covering a frequency range of 100 kHz to 0.01 Hz with a 10 mV (vs. OCP) AC perturbation amplitude. The test solutions were NaCl solutions with mass fractions of 1%, 3.5%, and 6%. EIS data were fitted using ZView3.1 software with the equivalent circuit model $R_{s}(Q_{1}(R_{f}(Q_{2}(R_{\mathrm{ct}}\;W))))$. All fitting parameters (including $R_{s}$, $Q_{1}$, $R_{f}$, $Q_{2}$, $R_{\mathrm{ct}}$, and the exponent values of constant phase elements) were recorded for subsequent analysis.

Mott–Schottky measurements were conducted at a fixed frequency of 1000 Hz, which is widely adopted in Mott–Schottky tests for steels [29,30,31,32,33] and thereby facilitates comparison and validation by researchers. The potential was scanned from −1 V to 1 V (vs. SCE) with a step potential of 10 mV.

## 3. Results and Discussion

### 3.1. Pitting Corrosion Behavior and Corrosion Product Evolution

**Figure 1:**
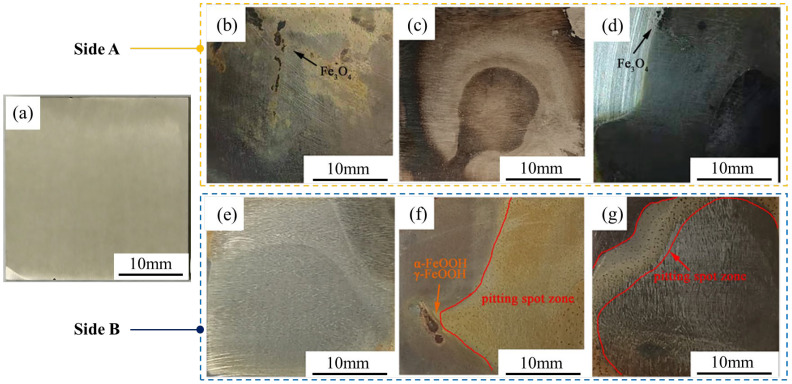
Macro-morphology of samples after 144 h of immersion in different chloride ion concentrations: (**a**) surface morphology of the sample before immersion (no pitting observed); (**b**–**d**) side A (facing the container opening); (**e**–**g**) side B (facing the container bottom); (**b**,**e**) 1% concentration, (**c**,**f**) 3.5% concentration, (**d**,**g**) 6% concentration.

Figure 1 presents the macroscopic morphology of the samples after 144 h of immersion. It can be observed that spotted corrosion pits have formed on the surface of all samples, which is consistent with the characteristics of pitting corrosion. Furthermore, as the chloride ion concentration increases, the dark spots (initial pitting sites) on the specimen surface become increasingly dense. A similar observation has been reported in [14]. With the increase in solute concentration, the severity of pitting corrosion in the affected areas intensifies, manifesting as more densely distributed corrosion pits. These phenomena indicate that high-concentration NaCl solutions can accelerate the corrosion of this material.

Throughout the corrosion process, it can be observed that easily detachable orange corrosion products first form on the material surface, then gradually transform into orange–red products, and finally black products tightly integrated with the material surface appear. Additionally, a layer of orange detached powder can be seen at the bottom of the container. Figure 1 reveals that numerous black attachments are present on side A (Figure 1b–d), while few black attachments are observed on side B (Figure 1e–g), suggesting that the black products are transformed from the orange ones. Consequently, it can be inferred that the corrosion process consists of two stages, each producing distinct corrosion products. As corrosion progresses, the easily detachable orange substances generated in the first stage gradually convert into the non-detachable black corrosion products formed in the second stage, leading to the gradual color change of the rust layer from orange to black. This viewpoint is consistent with the findings reported by Yamashita [34,35].

Therefore, the rust layer on the material surface can be divided into an inner rust layer predominated by orange corrosion products and an outer rust layer predominated by black products. X-ray diffraction (XRD) analysis was performed to determine their chemical compositions. The outer rust layer powder was collected from the solution precipitates after 144 h of immersion in 3.5% NaCl solution, while the inner rust layer powder was scraped from side A of the material under the same treatment conditions. Meanwhile, the rusted specimens (side A) with the rust layer retained were subjected to XRD scanning. The obtained XRD patterns are presented in Figure S2. Results indicate that the outer rust layer is mainly composed of α-FeOOH and γ-FeOOH, with a small amount of Fe_3_O_4_. In contrast, the inner rust layer consists of β-FeOOH, γ-FeOOH, and Fe_3_O_4_, where Fe_3_O_4_ accounts for the highest content, followed by β-FeOOH. γ-FeOOH is an orange substance and a thermodynamically metastable FeOOH, while α-FeOOH is an ochre-colored substance and a thermodynamically stable FeOOH. According to the Evans model [36,37], the following reaction relationships exist among α-FeOOH, γ-FeOOH, and Fe_3_O_4_:

(1) $$3\gamma -\mathrm{FeOOH}\;+\;H\;+\;e\;\to \;Fe_{3}O_{4}\;+\;2H_{2}O$$

(2) $$Fe^{2+}+2a-\mathrm{FeOOH}\;\to \;Fe_{3}O_{4}+2H^{+}$$

(3) $$3{\mathrm{Fe}}_{3}O_{4}+4/3O^{2-}+9/2H_{2}O\;\to \;9\alpha -\mathrm{FeOOH}$$

Therefore, at the initial stage of corrosion, in the presence of sufficient oxygen, Fe_3_O_4_ tends to react to form the more thermodynamically stable α-FeOOH [38]. As the corrosion layer thickens, the environment gradually becomes oxygen-deficient, which inhibits the transformation of Fe_3_O_4_ to α-FeOOH. In contrast, γ-FeOOH is highly unstable and prone to converting to Fe_3_O_4_; consequently, the corrosion products gradually evolve into a dense, black layer dominated by Fe_3_O_4_ [39,40]. Due to the formation of local anodic regions on the steel substrate surface, Cl^−^ ions in the solution are attracted by the positive charge of the anodic regions based on the principle of electromigration, thereby accumulating in the inner layer of the rust layer. This creates favorable conditions for the stable existence of β-FeOOH, and thus a relatively high content of β-FeOOH is detected in the inner rust layer [41].

**Figure 2:**
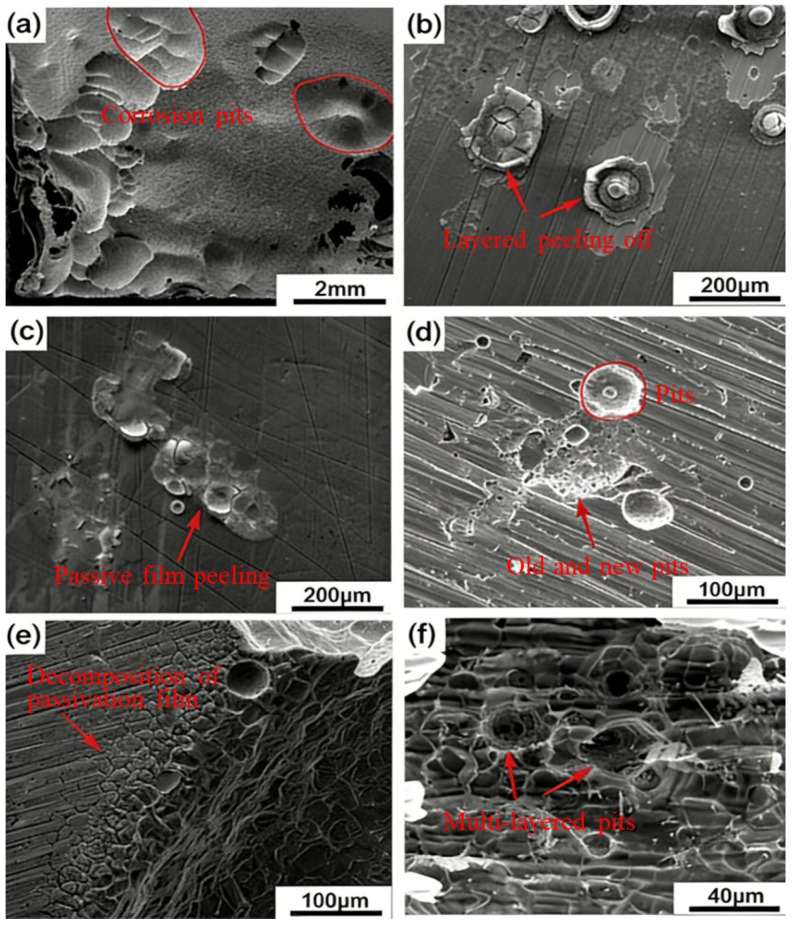
SEM images of samples after 144 h of immersion in 6% FeCl_3_ solution: (**a**–**c**) different regions of the same sample (magnification: 500×, 2000×, 5000×); (**d**–**f**) cross-sectional views of corrosion pits (magnification: 1000×, 2000×, 5000×), showing multi-layered pit structures.

To further analyze the corrosion behavior of 18%Ni high-strength steel, 6% FeCl_3_ solution (a standard accelerated pitting test medium) was used. Note that FeCl_3_ is more aggressive than NaCl (due to Fe^3^+ oxidizing the metal matrix), so results from FeCl_3_ cannot be directly compared to NaCl; this test only aims to verify the pitting propagation mechanism. From Figure 2a,b, it can be seen that the behavior of the steel is typical pitting corrosion. There are corrosion pits on the surface, and new corrosion pits will continue to extend in and around the old corrosion pits. The most important factor for pitting corrosion is the self-repair speed of the passive film [42,43]. In a chloride ion environment, the chloride ion is easy to pass and penetrates defects in the passive film due to the small radius to combine with the metal matrix. It makes the insoluble oxides on the surface of the steel turn into soluble chlorides, and the recovery speed of the passive film is severely reduced because the passivation state of the surface of the material is activated, resulting in pitting corrosion of the material [44,45,46]. Moreover, it can be observed that the residue of the passive film layer is peeling off, as shown in Figure 2b.

From Figure 2a,d,f, it can be observed that the corrosion pits are multi-layered, which indicates that the corrosion process proceeds layer by layer. First, the material will appear as small corrosion pits in a local area, and then under the further action of chloride ion, on the one hand, more small corrosion pits will appear and gradually connect into one piece; on the other hand, the small corrosion pits will gradually expand into the interior of the material. The chloride ion in the environment is constantly adsorbed on the pores and cracks on the surface of the material. Figure 2c shows the peeling of the passive film, which is attributed to Cl^−^ adsorption on the film defects and subsequent dissolution of the oxide layer (forming soluble metal chlorides) [47,48,49]. Figure 2b,c demonstrate that the membrane structure on the surface of the material is destroyed in the chloride ion medium, and then the material matrix is exposed [50]. In Figure 2e, we can see that the damage on the surface of the material mainly occurs at the defect of the material. New pitting pits are caused by the destruction of the passive film, and when they are enlarged to a certain size, the matrix that exposed the solution continues to adsorb chloride ions, resulting in the generation of new pitting pits. This cycle continues until the material is completely corroded.

**Figure 3:**
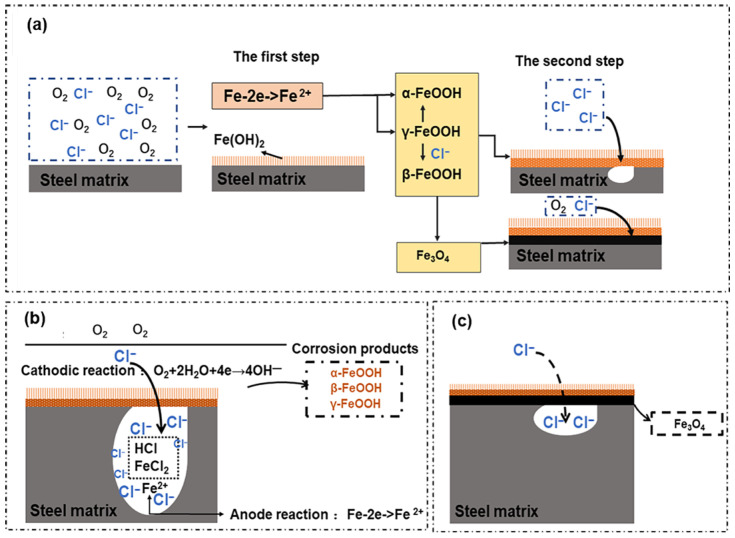
Schematic diagram of pitting corrosion mechanism of martensitic high-strength steel: (**a**) shows the corrosion mechanism of the material surface, (**b**) demonstrates the corrosion mechanism without dense corrosion products, and (**c**) illustrates the corrosion mechanism with dense corrosion products.

Figure 3 shows the corrosion mechanism of high-strength steel. From Figure 3a, it can be observed that in the corrosive medium, the chloride ion is firstly adsorbed on the surface of the material to generate highly soluble chloride, which is a corrosion product, to replace the oxide layer on the surface of the material. It corresponds to the production of the orange easily falling off of the material in the macroscopic phenomenon [33,36]. The passive film is difficult to repair, and then pitting occurs; after pitting, the chloride ion continues to invade further inside of the matrix. The hydrolysis reaction between the chloride ion and the matrix causes the pH of the solution in the pitting pores to drop, forming a strong acid solution area [51,52,53], which accelerates the dissolution of the metal and causes the pitting pits to expand and deepen. The metal ions in the pitting pit continue to increase. Under the action of the electric field, the chloride ion outside of the pit continuously moves into the pitting hole, the concentration of chloride ions in the pit continues to increase, and the pit continues to expand in and around until the steel fails [49,51], which is shown in Figure 3b. As the corrosion time increases, the corrosion products gradually become dense and cover the surface of the material, and the aggressive anions in the medium are isolated from the matrix. Therefore, the degree of corrosion decreases, as shown in Figure 3c.

### 3.2. Electrochemical Impedance Spectroscopy

**Figure 4:**
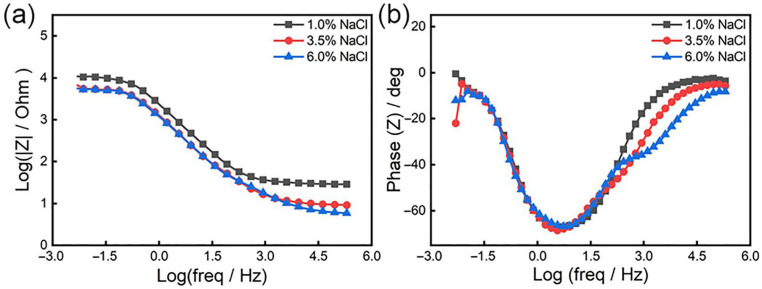
Bode diagram of 18%Ni high-strength steel under different chloride ion concentrations: (**a**) impedance–frequency; (**b**) phase angle–frequency.

**Figure 5:**
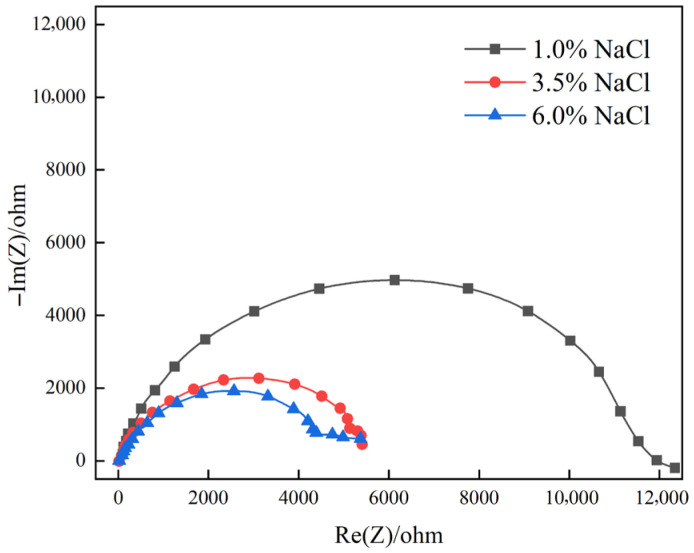
Nyquist spectra of 18%Ni high-strength steel under different chloride ion concentrations.

Figure 4 illustrates the Bode diagram of the samples at 1%, 3.5%, and 6% chloride ion concentrations, and Figure 5 illustrates the Nyquist spectra of the samples at different solutions. As shown in Figure 4, in the Bode curve, the phase angle of steel changes from small to large and then decreases from high-frequency to low-frequency, and there is a maximum phase angle (as shown in Figure 4b). With the increase of chloride ion concentration, the maximum phase angle of the Bode diagram gradually decreases, as well as the minimum phase angle at 6% concentration. This indicates that the dispersion effect is enhanced, the surface roughness of the steel increases [54,55,56], and the increase in roughness makes the chloride ion more easily adsorbed. Accordingly, we can conclude that on the surface of the material, the instability of the passive film is increased, and corrosion occurs more easily. From Figure 4b, there are two-time constants at a concentration of 1% and 3.5%, while at a chloride ion concentration of 6%, the Bode diagram has three peaks, which indicates that at this concentration, the test has three-time constants [57].

Figure 5 shows the impedance spectra of different concentrations of chloride ions. From Figure 5, we can see that the high-frequency part of the sample impedance spectrum in the medium of 1% chloride ion concentration is a capacitive reactance arc, and there is an inductive reactance near the low frequency, which is where the low-frequency data enter the fourth quadrant. This part indicates that the sample has pitting corrosion at this concentration induction period [58,59,60]. As the concentration increases, the low-frequency inductive reactance characteristics in the Nyquist spectrum at 3.5% chloride ion concentration and 6% chloride ion concentration disappear and are replaced by a low-frequency capacitive reactance arc, which means the pitting corrosion enters a state of stable growth. Hence, as the concentration increases, the corrosion of the high-strength steel becomes more serious, and the corrosion resistance is gradually decreasing.

From Figure 5, we can see that the Nyquist spectrum of steel at a chloride ion concentration of 3.5% has two obvious capacitive impedance arcs, while the sample at a chloride ion concentration of 6% has three obvious capacitive impedance arcs. A capacitive reactance arc is in the high-frequency region and another capacitive reactance arc is in the middle- and low-frequency region. The capacitive reactance arc in the low-frequency region is related to the capacitance of the electric double layer, and the capacitive reactance arc in the high-frequency region is related to the passive film [59,61]. The disappearance of the inductive arc in the Nyquist spectrum demonstrates that the pitting corrosion has grown steadily; the appearance of the capacitive arc in the low-frequency region under a concentration of 6% reveals that the surface of the sample has produced a dense substance, which insulates the contact between the material and the solution. As the corrosion cycle increases, dense materials will be produced on the surface of the material to protect the matrix from corrosion [33,61,62,63].

The diameter of the high-frequency semicircle in the Nyquist spectrum is directly proportional to the corrosion resistance of the material. A larger diameter indicates better corrosion resistance, as it reflects higher impedance of the passive film and slower charge transfer at the metal–solution interface. According to Figure 5, as the concentration increases, the diameter of the high-frequency semicircle of steel gradually decreases. When the chloride ion concentration is 6%, the capacitive reactance arc diameter in impedance spectroscopy is the smallest, indicating that as the chloride ion concentration increases, the corrosion rate increases [59].

**Figure 6:**
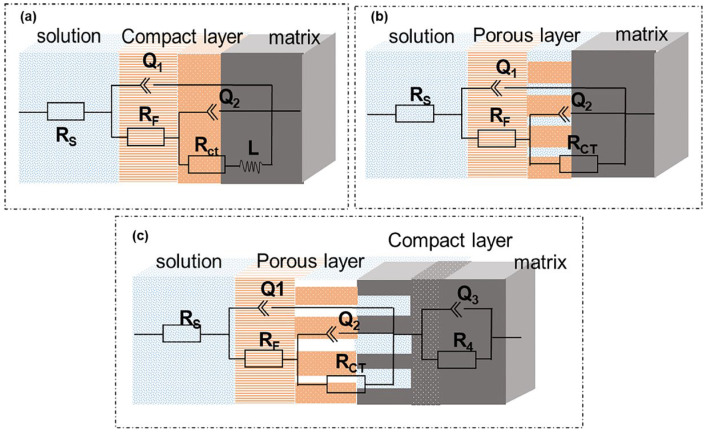
Equivalent circuit diagram of martensitic high-strength steel in different chloride ion media: (**a**) 1% Cl concentration, (**b**) 3.5% Cl concentration, (**c**) 6% Cl concentration.

Figure 6 is the equivalent circuit diagram of fitting EIS under different chloride ion concentrations, where (a) is the equivalent circuit diagram under 1% chloride ion concentration using $R_{s}\;+\;Q_{1}/(R_{f}\;+\;Q_{2}/(R_{\mathrm{ct}}\;+\;L))$ method fitting, (b) is the equivalent circuit diagram at 3.5% chloride ion concentration using the $R_{s}\;+\;Q_{1}/(R_{f}\;+\;Q_{2}/R_{\mathrm{ct}})$ method to fit, and (c) is the equivalent circuit diagram at 6% chloride ion concentration using $R_{s}\;+\;Q_{1}/(R_{f}\;+\;Q_{2}/R_{\mathrm{ct}})\;+\;Q_{3}/R_{4}$ for fitting. Among them, $R_{s}$ represents the resistance of the solution, $R_{f}$ is the product resistance on the electrode surface, $R_{\mathrm{ct}}$ is the charge transfer resistance, $R_{4}$ is the resistance of the adsorbed substance, $Q_{1}$ is the electric double layer between the solution and the membrane, and $Q_{2}$ is the electric double layer produced by the solution and the material matrix. $Q_{3}$ is the electric double layer produced by the solution and the adsorbed substance at a concentration of 6%; L corresponds to the parameters of the inductive reactance part in Figure 5.

The electrochemical corrosion parameters of the steel under different chloride ion concentrations are obtained through fitting, as shown in Table 1. The polarization resistance $R_{p}\;=\;R_{f}\;+\;R_{\mathrm{ct}}$ is defined here. Table 1 reveals that as the chloride ion concentration increases, the dispersion index “*n*” value continues to decrease, indicating that the surface of the passive film becomes looser and looser, the surface defects of the steel become larger and larger, and corrosion proceeds more easily. Consistently, the value of polarization resistance gradually decreases with the increase in chloride ion concentration. When the polarization resistance is smaller, the corrosion rate is faster, so when the chloride ion concentration is 6%, the $R_{p}$ value is the lowest and the corrosion behavior of steel is the strongest.

### 3.3. Polarization Curve

**Figure 7:**
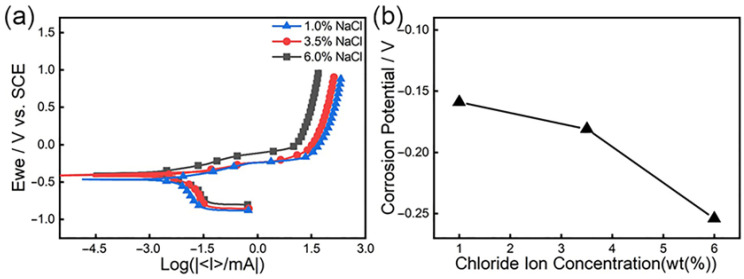
Polarization curves of 18%Ni high-strength steel under different chloride ion concentrations: (**a**) polarization curve; (**b**) corrosion potential and chloride ion concentration.

Figure 7 is the polarization curve of 18%Ni high-strength steel under different chloride ion concentrations. From Figure 7a, we can reveal that as the potential rises, there will be a sudden change point at a certain potential, and then as the potential gradually increases, the flow density further increases, and the corrosion rate will increase. Now, the corresponding potential is the corrosion potential, which can reflect the difficulty with which the passive film is destroyed. The higher the corrosion potential, the better the corrosion resistance of steel, and the lower the corrosion potential, the worse the corrosion resistance of steel [64,65]. Figure 7b is a graph of the change of corrosion potential under different concentrations. It is obvious from the figure that the corrosion potential of the mutation point at 1% concentration is the highest, and the mutation point potential at 6% concentration is the lowest, indicating that as the chloride ion concentration increases, the test of the corrosion potential drops, and corrosion is more likely to occur.

As the potential further increases, the current increase becomes slower and the anode reaction is inhibited due to the adsorption of corrosion products on the surface of the material, as mentioned above [33,66], causing the substrate to be isolated from the solution, which results in the corrosion rate of steel decreasing.

**Figure 8:**
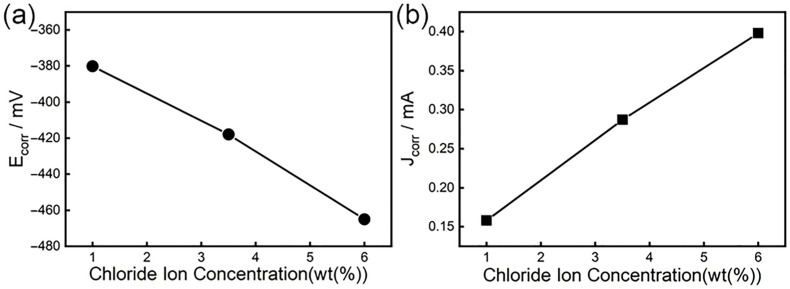
Changes of self-corrosion potential and self-corrosion current under different chloride ion concentrations: (**a**) the relationship between $E_{\mathrm{corr}}$ and chloride ion concentration; (**b**) the relationship between $J_{\mathrm{corr}}$ and chloride ion concentration.

Figure 8 shows the relationship between the self-corrosion potential and corrosion current density of high-strength steel and the chloride ion concentration. Figure 8a indicates that the self-corrosion potential ($E_{\mathrm{corr}}$) increases with the chloride ion concentration. When the chloride ion concentration is 6%, $E_{\mathrm{corr}}$ is the lowest. Now, the corrosion resistance of steel is the worst, but $E_{\mathrm{corr}}$ cannot be used as a standard for the corrosion resistance of materials alone. According to Farady’s second law, if the amount of electricity passed is the same, the chemical equivalents of different substances precipitated or dissolved are the same, that is, the corrosion current density is proportional to the corrosion rate [67,68,69]. Figure 8b reflects the relationship between the corrosion current density ($J_{\mathrm{corr}}$) of steel and the Cl^−^ concentration. It is clear that as the chloride ion concentration increases, $J_{\mathrm{corr}}$ shows a trend of increasing. At 1% chloride ion concentration, the $J_{\mathrm{corr}}$ value is the smallest and the corrosion rate is the smallest; at 6% chloride ion concentration, the $J_{\mathrm{corr}}$ value is the largest and the corrosion rate is the largest. This shows that as the chloride ion concentration increases, the corrosion resistance of steel decreases.

Studies have found that the semiconductor properties of the passive film are closely related to its corrosion resistance [70,71]. Through the study of the semiconductor properties of the passive film, we can understand the difficulty of ion transfer in the passive film and then infer the protective effect of the passive film on the substrate. The principle is that when the film is in contact with the solution, the semiconductor film and the solution have opposite charges, and the excess charge of the semiconductor film is distributed in the space charge layer [72,73,74]. When an external voltage causes the space charge to show the depletion layer, the relationship between the space charge capacitance and the potential obeys the Mott–Schottky equation:

For n-type semiconductor films, the relationship between space charge capacitance (*C*) and potential (*E*) is as follows:

(4) $$C_{\mathrm{interface}}^{-2}\;={\;C}_{\mathrm{SC}}^{-2}\;=\;\frac{2}{\varepsilon \varepsilon_{0}eN_{D}}\left(E\;-{\;E}_{\mathrm{fb}}\;-\;\frac{\mathrm{kT}}{e}\right)$$

For p-type semiconductors, the relationship between space charge capacitance (*C*) and potential (*E*) is as follows:

(5) $$C_{\mathrm{interface}}^{-2}\;={\;C}_{\mathrm{SC}}^{-2}\;=\;-\frac{2}{\varepsilon \varepsilon_{0}eN_{A}}\left(E\;-\;E_{\mathrm{fb}}\;-\;\frac{\mathrm{kT}}{e}\right)$$

where

$C_{\mathrm{interface}}$ = interface capacitance (F·cm^−2^)

$C_{\mathrm{SC}}$ = space charge layer capacitance (F·cm^−2^)

ε = dielectric constant of the passive film

$\varepsilon_{0}$ = vacuum permittivity (8.854 × 10^−14^ F·cm^−1^)

e = elementary charge (1.602 × 10^−19^ C)

$N_{D}$ = donor density (cm^−3^),

$N_{A}$ = acceptor density (cm^−3^)

E = applied potential (V vs. SCE)

$E_{\mathrm{fb}}$ = flat band potential (V vs. SCE)

k = Boltzmann constant (1.381 × 10^−23^ J·K^−1^)

T = absolute temperature (298 K)

**Figure 9:**
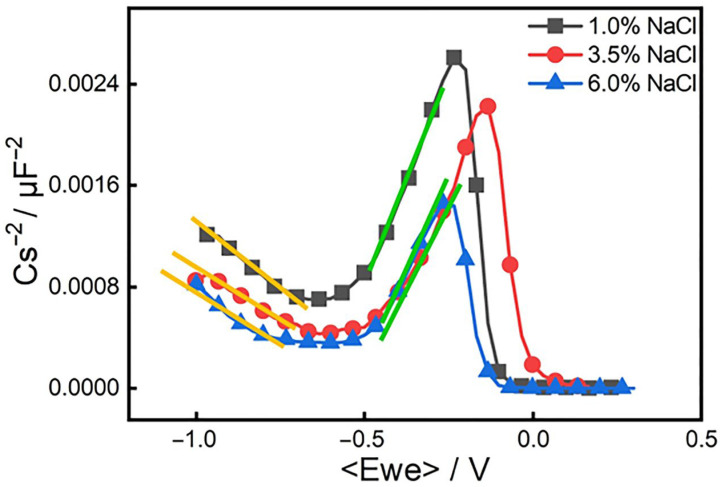
Mott–Schottky curve of 18%Ni high-strength steel under different chloride ion concentrations. The yellow and green lines represent linear fitting lines of the Mott–Schottky curves in specific potential intervals: the yellow line fitting result is used to calculate the acceptor density ($N_{A}$); the green line fitting data is used to determine the donor density ($N_{D}$).

Figure 9 shows the Mott–Schottky curve of the passive film of steel under different chloride ion concentration conditions. Figure 9 points out that the trends of the Mott–Schottky curves under the three concentrations are basically the same, and the curves are all linear. The passive film of steel exhibits different semiconductor types in different potential ranges. According to research, when Fe oxides accumulate, the space charge layer is in an enriched state, while the space charge layer of other oxides is in a depleted state. In the dead state, the measurement result of the passive film capacitance is a p-type semiconductor; otherwise, it is an n-semiconductor. The slope of the curve of the steel in the solution in the potential interval lower than the flat band potential is negative, indicating that the semiconductor properties of the passive film in this potential interval are p-type semiconductors. The slope of the curve in the potential interval that is higher than the flat band potential is positive, and the semiconductor properties of the passive film are n-type semiconductors. The n–p semiconductor conversion interval of steel is between −0.7 and −0.5 V, which is consistent with the corrosion potential fitted by the polarization curve. The slope of the Mott–Schottky curve of the passive film of steel at different concentrations is different.

**Figure 10:**
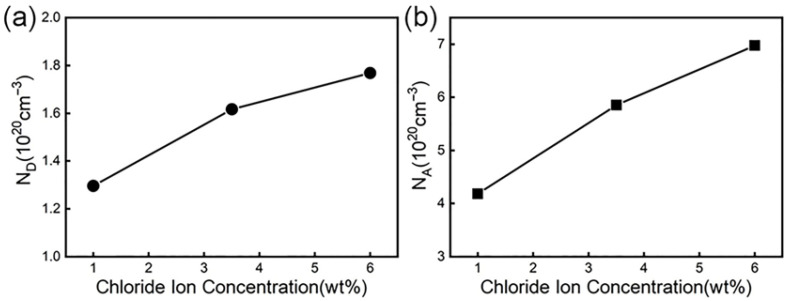
Curves of donor density and acceptor density under different chloride ion concentrations: (**a**) the curve of donor density versus chloride ion concentration; (**b**) electric change curve of acceptor density with chloride ion concentration.

It can be seen from the Equations (1) and (2) that the donor density and acceptor density can be obtained through the linear part in Figure 9, that is, the slope of the curve of the straight part. Figure 10 is a graph showing the change of donor concentration and acceptor concentration with chloride ion concentration. From the point defect model (PDM), we see that there is a dynamic equilibrium between the growth of the passive film at the metal substrate/film interface and the dissolution of the passive film at the film/electrolyte interface. When the passive film is in a solution containing corrosive ions, the oxygen vacancies at the membrane/electrolyte interface can adsorb corrosive ions and react with them to generate oxygen vacancies and metal ion vacancies. The generated oxygen vacancies can interact with other reactions at the membrane/solution interface to generate more metal ion vacancies [75,76]. These oxygen vacancies and metal ion vacancies as donor densities are reflected in the Mott–Schottky equation. Therefore, the donor current density reflects the number of point defects in the space charge layer.

From Figure 10a, we can see that with the increase in chloride ion concentration, the donor density is continuously increasing. The reason is that with the increase in the solution concentration in the high-concentration solution, the diffusion layer of the solution becomes thinner, thus accelerating the growth rate of the film, but the time for atoms or ions to reach the “correct” position is relatively short, so more defects will be produced in the passive film and the donor concentration will be correspondingly larger. The greater the donor current density, the greater the number of point defects in the space charge layer, and the damage to the film first occurs at these defects. Therefore, as the chloride ion concentration increases, the probability of damage to the passive film will increase. When the passive film is destroyed and the self-repair speed of the passive film is less than the defect generation speed, pitting corrosion occurs.

Through Figure 10b, we can see that the acceptor density also shows an increasing trend with the increase in chloride ion concentration. This is mainly because the chloride ion concentration continues to rise, which makes the number of anions adsorbed on the surface of the oxide film formed by steel continue to increase, which in turn generates soluble metal chlorides, resulting in an increase in metal cations [70,77,78]. The increase in cations will make the corrosion potential smaller, thus speeding up the corrosion of the material. Therefore, the chloride ion enables the material to be more susceptible to corrosion by making the passive film of the material extremely unstable, thereby decreasing the protection performance of the film.

**Figure 11:**
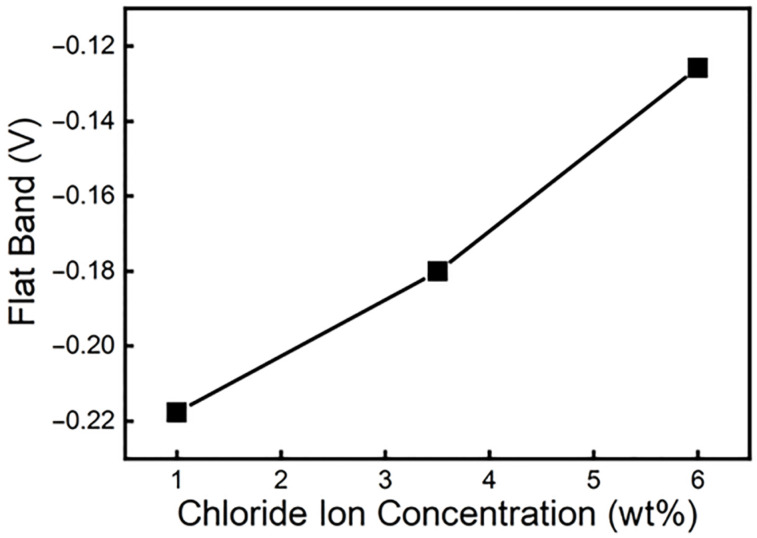
Flat band potential distribution diagram under different chloride ion concentrations.

The flat band potential of a material is inversely proportional to the level of the Fermi level of the material, and the level of the Fermi level reflects the ability of the material to gain and lose electrons. When the flat band potential increases, the Fermi energy level will decrease, which signifies that the ability to gain electrons increases, which increases the difficulty of forming the passive film. Therefore, the defects in the passive film increase. The above makes the self-repairing speed of the passive film slow down and weakens the corrosion resistance of the material. According to calculations, the flat band potentials at chloride ion concentrations of 1%, 3.5%, and 6% are −0.2177 V, −0.1801 V, and −0.1258 V, respectively. Figure 11 is a graph of the flat band potential changes under different chloride ion concentrations. This reveals more intuitively from Figure 11 that the flat band potential gradually increases with the chloride ion concentration. Then, the passive film becomes difficult to form, and the corrosion rate accelerates. In other words, chloride ions reduce the corrosion resistance of the material by affecting the stability of the passive film’s surface.

## 4. Conclusions

The effects of chloride ion concentration on the corrosion behavior of martensitic high-strength steel (00Ni18Co8Mo5TiAl) were systematically investigated in this work. The main conclusions can be drawn as follows:

(1) In chloride environments, 18%Ni high-strength steel exhibits distinct pitting corrosion, with initial corrosion pit density increasing significantly with chloride concentration (sparse at 1% to dense at 6%). Corrosion propagates layer by layer, as initial micro-pits (≤200 μm) form first and then expand inward and coalesce into large pits (≥500 μm) via continuous chloride infiltration. Corrosion products undergo a quantitative transition, as outer orange α-FeOOH/γ-FeOOH (~60% content) converts to inner dense Fe_3_O_4_/β-FeOOH (Fe_3_O_4_ accounting for ~55%), validated through XRD.
(2) Electrochemical characterization shows a degradation of corrosion resistance with rising chloride concentration, as polarization resistance ($R_{p}\;=\;R_{f}\;+\;R_{\mathrm{ct}}$) decreases from 2.93 × 10^5^ Ω/cm^2^ (1%) to 1.21 × 10^5^ Ω/cm^2^ (6%), corrosion current density ($J_{\mathrm{corr}}$) increases from 0.15 mA/cm^2^ to 0.35 mA/cm^2^, and self-corrosion potential ($E_{\mathrm{corr}}$) shifts negatively from −0.38 V to −0.48 V vs. SCE. Furthermore, during the corrosion process, the dense corrosion products formed on the surface of the material play the role of protecting the matrix in the subsequent corrosion process.
(3) The passive film exhibits potential-dependent semiconductor behavior, with p-type (negative Mott–Schottky slope) below the flat band potential ($E_{\mathrm{fb}}$) and n-type (positive slope) above $E_{\mathrm{fb}}$. The n–p conversion interval is −0.7 V to −0.5 V vs. SCE, overlapping with the corrosion potential range (−0.38 V to −0.48 V), confirming a direct correlation with corrosion activity.
(4) Mott–Schottky analysis reveals chloride-induced defects in the passive film. $E_{\mathrm{fb}}$ increases linearly from −0.2177 V (1%) to −0.1258 V (6%), while donor density ($N_{D}$) and acceptor density ($N_{A}$) rise by ~60% and ~45% at 6% vs. 1%. When in a high concentration of chloride ion medium, the passive film on the steel surface becomes unstable, which causes the steel to be more susceptible to corrosion, and the corrosion rate increases.

## Figures and Tables

**Table 1:** Equivalent circuit parameters of steel in different chloride ion concentrations.

Chloride ion concentrations (wt.%)	1	3.5	6
$R_{s}$ (Ω/cm^2^)	34.7	19.3	11.8
$Q_{1}$ (F/cm^2^)	2.13 × 10^−5^	2.47 × 10^−5^	2.80 × 10^−5^
$n_{1}$	0.7906	0.7206	0.6943
$R_{f}$ (Ω/cm^2^)	2.35 × 10^5^	9.18 × 10^4^	8.79 × 10^4^
$Q_{2}$ (F/cm^2^)	1.82 × 10^−5^	2.18 × 10^−5^	2.47 × 10^−5^
$n_{2}$	0.778	0.712	0.683
$R_{\mathrm{ct}}$ (Ω/cm^2^)	5.84 × 10^4^	3.82 × 10^4^	3.29 × 10^4^
L (H·cm^2^)	1.17 × 10^3^	/	/
$Q_{3}$ (F/cm^2^)	/	/	2.98 × 10^−5^
$n_{3}$	/	/	0.667
$R_{4}$ (Ω·cm^2^)	/	/	7.43 × 10^4^

## Data Availability

The original contributions presented in this study are included in the article. Further inquiries can be directed to the corresponding authors.

## Supplementary Materials

The following supporting information can be downloaded at https://www.mdpi.com/article/10.3390/ma19020444/s1, Figure S1: Schematic diagrams of corrosion performance testing devices: (a) Chemical immersion test; (b) Electrochemical test; Figure S2. XRD scanning results of the outer rust layer and inner rust layer of the sample after corrosion in 3.5%NaCl solution for 144 h.

## Abbreviations

The following abbreviations are used in this manuscript:

EIS	Electrochemical Impedance Spectroscopy
SEM	Scanning Electron Microscope
SCE	Saturated Calomel Electrode
RE	Reference Electrode
CE	Counter Electrode
M-S curve	Mott–Schottky curve
PDM	Point Defect Model
