# Supplementary material for: Chloride Ion-Induced Modification of Passive Film on the Surface of 18%Ni High-Strength Steel
Source: Materials (Basel). 2026 Jan 22;19(2):444. doi: 10.3390/ma19020444 (PMC12843305; doi:10.3390/ma19020444)
Supplement: Supplementary file 1 [file materials-19-00444-s001.zip › materials-4086660-supplementary.pdf]

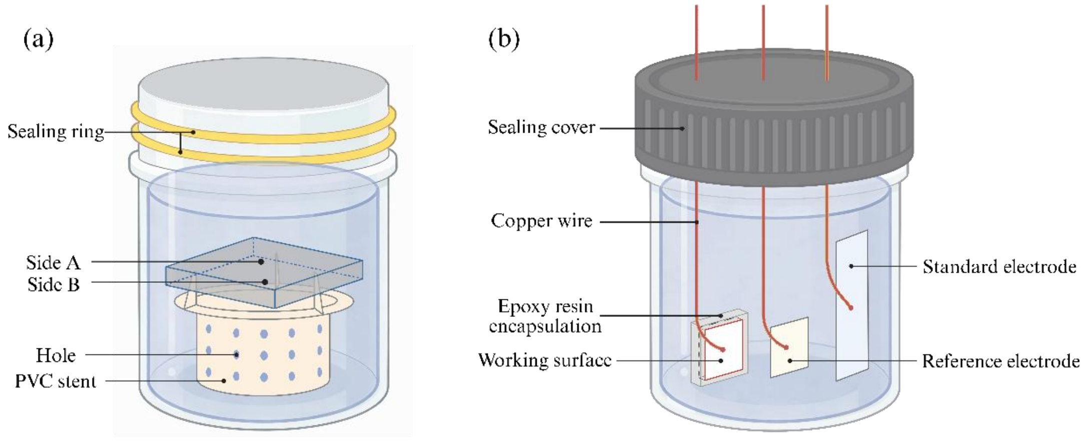

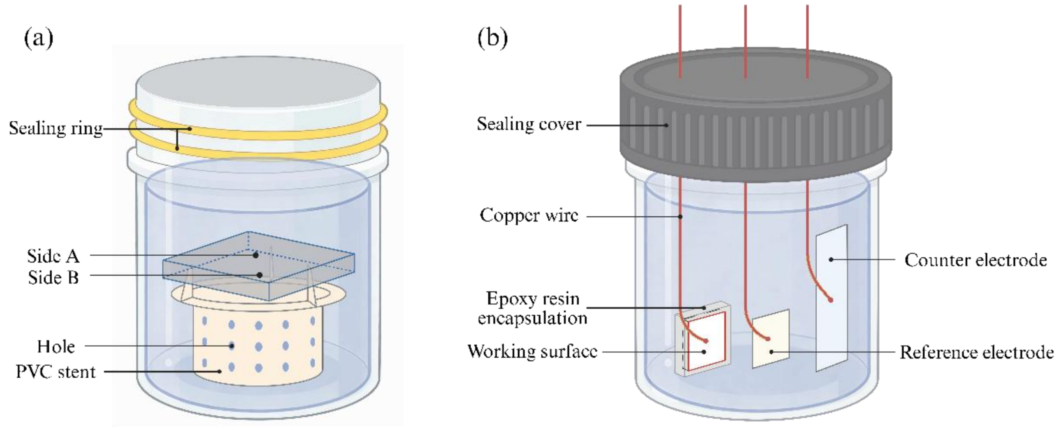

Figure S1. Schematic diagrams of corrosion performance testing devices: (**a**) Chemical immersion test; (**b**) Electrochemical test.

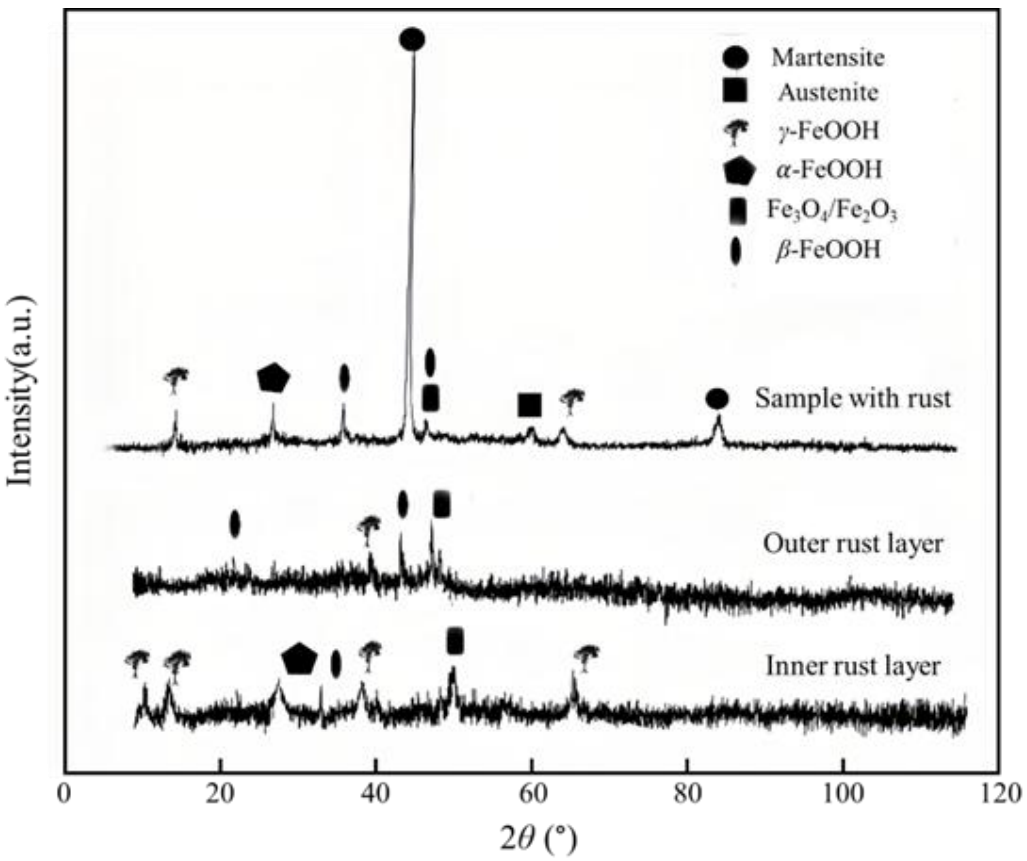

Figure S2. XRD scanning results of the outer rust layer and inner rust layer of the sample after corrosion in 3.5%NaCl solution for 144 hours.
